# Response to: “best practices when benchmarking CATCH for the design of genome enrichment probes”

**DOI:** 10.1093/bioinformatics/btag100

**Published:** 2026-03-03

**Authors:** Jarno N Alanko, Ilya B Slizovskiy, Daniel Lokshtanov, Travis Gagie, Noelle R Noyes, Christina Boucher

**Affiliations:** Department of Computer Science, University of Helsinki, 00014 Helsinki, Finland; College of Veterinary Medicine, Purdue University, West Lafayette, IN 47907, United States; Department of Computer Science, University of California, Santa Barbara, CA 93106, United States; Faculty of Computer Science, Dalhousie University, Halifax, NS, B3H 4R2, Canada; Department of Veterinary Population Medicine, University of Minnesota, St. Paul, MN 55108, United States; Department of Computer and Information Science and Engineering, University of Florida, Gainesville, FL 32611, United States

## Abstract

We clarify the design principles and evaluation choices underlying Syotti, a robust and scalable probe-design tool developed to support large, heterogeneous bacterial datasets with minimal parameter tuning. We highlight Syotti’s ability to perform simultaneous large-scale designs and its effectiveness as a reliable alternative when existing tools such as CATCH are not well suited to the problem setting.

We appreciate the opportunity to respond to the points Metsky et al. (2026) raised regarding our work on Syotti (Alanko *et al.* 2022). Our primary objective in designing Syotti was to create a straightforward, robust, publicly available tool capable of handling a variety of input types with minimal free parameters. While Metsky *et al.* (2019)’s CATCH advocates for independently designing probe sets for different taxa and then pooling them together, this approach is not suitable for a bacterial dataset we work with that contains only a few genera and numerous serotypes and was the primary use case around which Syotti was designed. We note that even a small increase in the number of probes can push a design into a higher manufacturing tier, substantially increasing costs for large probe sets.

As we stated in our original paper, “CATCH was designed such that large datasets should be separated by strain or substrain into smaller datasets, have the baits designed on each smaller dataset, and then be combined into a bait set for the complete dataset. This appears to work well if…the data can be separated into small subsets but does not work so well for whole bacterial genomes or datasets that cannot be separated into small subsets by strain information.” When the dataset is not separated at all and presented as a single input sequence, Metsky *et al.* note in their supplementary material that “CATCH v1.4.0…crashes due to a bug.” Thus, an algorithm capable of handling large designs simultaneously is required, and we consider this one of Syotti’s key contributions over CATCH.

Below is our detailed response to each issue raised:

## Issue (1): clarification of specific options

The CATCH README file from 26 January 2021 lists ten options for the design.py script, including—filter-with-lsh-hamming, which was not then explicitly recommended but noted as potentially reducing runtime and memory usage. In a December 2021 email exchange, Metsky *et al.* did not mention the—filter-with-lsh-minhash flag when we asked about the parameters to use, although they now recommend it.

Optimizing the values for the ten options that CATCH provides would require a grid search across the full parameter space, which is out of scope for any reasonable comparison of bioinformatics software. We also note the following from the original CATCH paper:

- “CATCH uses locality-sensitive hashing (LSH) to reduce the number of candidate probes, which is particularly useful when the input is a large number of highly similar sequences.” (This does not apply to our case, as our use case involved a large number of highly dissimilar sequences.)
- “Use of LSH to reduce the number of candidate probes is optional in our implementation of CATCH; we did not use it to produce the probe sets in this work.”

## Issue (2): reduction in probe-based enrichment efficiency

Metsky *et al.* did not provide a reference to support their claim of a “well-established reduction in probe-based enrichment efficiency,” nor did they cite a standard mismatch threshold. In our work, we conducted numerous probe capture experiments using mismatch tolerances greater than 30, consistent with the manufacturer’s guidance. As we reported in our paper, “As advised by the bait manufacturer Agilent, we set the bait length to 120 and tolerated 40 mismatches.”

## Issue (3): parameter choices and benchmarking

At the time of our experiments, the CATCH README stated, “While design.py requires particular choices of parameter values, pool.py is a program to find optimal hybridization parameters that can vary across many inputs…You need to run design.py on each dataset over a grid of parameter values that span a reasonable domain…We recommend running this multiple times and selecting the output that has the smallest loss.” This process raises several benchmarking challenges: it is unclear how to define a reasonable domain, how dense the grid should be, or how to balance runtime against probe quality. Reporting runtime only after parameter optimization does not reflect the full computational cost; if parameter search is essential, that cost should be included.

We experimented with optional parameters such as -cluster-and-design-separately 0.15 and -cluster-from-fragments 10000, as documented, but observed no significant improvement in runtime or results. We did not experiment with -filter-with-lsh-minhash, which we explicitly acknowledge in our paper. Although grid search could potentially improve Syotti’s runtime as well, we intentionally used fixed default parameters across all datasets to avoid overfitting. In contrast, Metsky *et al.* seem to have applied parameter tuning only to CATCH and not to Syotti, undermining one of their main points. Also, their original publication did not compare against existing tools such as *BaitFisher* (Mayer *et al.* 2016), despite its availability at the time.

## Issue (4): virus dataset parameters

We attempted to use identical parameters for Syotti and CATCH on the viral dataset, but CATCH did not complete within the reported time frame. These results are shown in Table 3 of our paper. To place CATCH in the best possible position for coverage analysis, we therefore used the bait sets Metsky *et al.* designed and published. Table 1, available as supplementary data at *Bioinformatics* online in their paper, shows species-specific overhang lengths, making it unclear which value should be used. The most common overhang value reported is zero, which is also the value assumed in Syotti’s mathematical formulation. Therefore, we chose this value as the most appropriate for a head-to-head comparison of coverage. Our reported 84% CATCH coverage is therefore not an error but an independent evaluation under a no-overhang coverage model.

## Issue (5): probe coverage model

Our mathematical problem statement explicitly requires coverage of every position in the input dataset and thus does not include overhang. While incorporating adjacent coverage could reduce the number of probes, we prioritized strict positional coverage. Extending Syotti to support alternative coverage models is possible, but we adhered to the requirements defined in our formulation.

In conclusion, we are pleased to see usability improvements in the newer versions of CATCH and believe that they will benefit users. We note that a related patent application declared in the original CATCH paper has since been granted (US11332783B2). In cases where CATCH does not scale to user needs, we continue to recommend Syotti as an alternative design strategy.

## Author contributions

Jarno N. Alanko (Conceptualization, Methodology, Software, Investigation, Formal analysis, Writing—original draft, Writing—review & editing), Ilya B. Slizovskiy (Investigation, Writing—review & editing), Daniel Lokshtanov (Writing—review & editing) Travis Gagie (Writing—review & editing), Noelle R. Noyes (Investigation, Writing—review & editing), Christina Boucher (Conceptualization; Project administration, Writing—review & editing).

## Conflicts of interest

None declared.

## Funding

None declared.
